# *Astragalus propinquus* schischkin and *Salvia miltiorrhiza* bunge promote angiogenesis to treat myocardial ischemia *via* Ang-1/Tie-2/FAK pathway

**DOI:** 10.3389/fphar.2022.1103557

**Published:** 2023-01-09

**Authors:** Mu-Xin Zhang, Xue-Ying Huang, Yu Song, Wan-Li Xu, Yun-Lun Li, Chao Li

**Affiliations:** ^1^ First Clinical Medical College, Shandong University of Traditional Chinese Medicine, Jinan, China; ^2^ College of Pharmacy, Shandong University of Traditional Chinese Medicine, Jinan, China; ^3^ Innovative Institute of Chinese Medicine and Pharmacy, Shandong University of Traditional Chinese Medicine, Jinan, China; ^4^ College of Traditional Chinese Medicine, Shandong University of Traditional Chinese Medicine, Jinan, China

**Keywords:** angiogenesis, myocardial ischemia, pericyte recruitment, astragalus propinquus, Salvia miltiorrhiza

## Abstract

*Astragalus propinquus* Schischkin and *Salvia miltiorrhiza* Bunge (AS) have been clinically used as adjunctive drugs in the treatment of myocardial ischemia (MI). However, the effect and mechanism of AS on MI have yet to be fully recognized. Here, we explored the cardioprotective effect of their combined use, and the mechanism of promoting angiogenesis through pericyte recruitment. Our data revealed that AS reduced MI and protects cardiac function. AS-treated MI mice exhibited reduced ST-segment displacement and repolarization time, increased ejection fraction, and less BNP and NT-proBNP expression. Pathological studies showed that, AS reduced the area of infarcted myocardium and slowed down the progress of cardiac remodelling and fibrosis. In addition, AS increased the content of platelet-derived growth factor receptors β (PDGFR-β), platelet endothelial cell adhesion molecule-1 (CD31) and angiogenesis-related proteins including vascular endothelial cadherin (VE-cadherin), Vascular Endothelial Growth Factor (VEGF) and transforming growth factor β (TGF-β). Moreover, these botanical drugs upregulated the expression of Angiopoietin-1 (Ang-1), phosphorylated angiopoietin-1 receptor (p-Tie-2), focal adhesion kinase (FAK) and growth factor receptor bound protein 7 (GRB7), indicating that the cardioprotection-related angiogenesis effect was related to pericyte recruitment, which may be through Ang-1/Tie-2/FAK pathway. In summary, AS can treat MI by protecting cardiac function, attenuating cardiac pathological changes, and hindering the progression of heart failure, which is related to angiogenesis after pericyte recruitment. Therefore, AS at a certain dose can be a promising treatment for MI with broad application prospects.

## 1 Introduction

MI, a disease of the blood vessels that supply the heart muscle, occurs when blood flow through one or more of coronary arteries is decreased, preventing the heart muscle from receiving enough oxygen (Wang et al., 2013). According to the World Health Organization (WHO), MI is one of the top three causes of years of life lost due to premature death globally. Patients with MI should have access to appropriate technology and medication. However, the most widely used therapy for restoring blood supply after MI is also facing defects. The incidence of insufficient reflow in infarcted area after percutaneous coronary intervention (PCI) was 46%, and the MI of patients receiving coronary artery bypass graft (CABG) was only reduced by 30%. (Qiu et al., 2017; Doenst et al., 2019). Therefore, angiogenesis is a promising therapeutic strategy to partially restore myocardial perfusion.

Angiogenesis, the derivation of new capillaries from existing capillary venules, has the ability to improve blood supply to ischemic areas after MI (Fan et al., 2006; Bu et al., 2020). This therapeutic effect is inseparable from the action of pericytes, the elongated supporting cells of the blood vessel wall (Armulik et al., 2005). Under normal physiological conditions, pericytes are distributed along endothelial cells and inhibit excessive angiogenesis (Tang et al., 2017). Following MI, the myocardium is affected by hypoxia and glucose deprivation, resulting in the release of pericytes from the vessel wall (Teichert et al., 2017). Endothelial cells isolated from pericytes have the ability to proliferate and migrate under the action of VEGF-A from pericytes, thereby enabling the generation of new capillaries (Franco et al., 2011). In addition to the early stages of angiogenesis, pericyte recruitment is also involved in maintaining the stability of newly formed blood vessels. After a certain degree of angiogenesis, pericytes return to the vicinity of endothelial cells to cover the new blood vessels, thereby stabilizing the new blood vessels and promoting their maturation (Dobaczewski et al., 2004; Stratman et al., 2010). Futhermore, pericytes derived TGF-β to inhibit the immortal proliferation of endothelial cells (Winkler et al., 2010). Therefore, therapeutic angiogenesis plays a crucial role in the treatment of MI. Unfortunately, there is a lack of cost-effective, controllable, and reproducible methods to promote angiogenesis by modulating pericyte recruitment. In this context, botanical drugs may be a proven complementary and alternative therapy.

Botanical drugs have been used clinically for more than 5,000 years with a systematic theoretical basis that guarantees their efficacy. Notably, a growing number of studies have shown that many natural medicines are powerful inducers of angiogenesis (Bernas, 2003). *Astragalus propinquus* Schischkin [*Leguminosae*; Astragalus membranaceus radix et rhizoma] and *Salvia miltiorrhiza* Bunge [*Lamiaceae*; Salviae miltiorrhizae radix et rhizoma] are both one of the most popular botanical drugs in the world, they can also be used as a drug pair to improve blood circulation, protect ischemia-reperfusion injury, and improve cardiac function in mice with MI (Li et al., 2008; Ma et al., 2013; Fu et al., 2014; Li et al., 2018). Accumulating evidence suggests that *Astragalus propinquus* Schischkin and *Salvia miltiorrhiza* Bunge can play a protective role against MI. However, their combined effect of promoting angiogenesis by regulating pericyte recruitment is yet to be elucidated. In this paper, a left anterior descending coronary artery ligation (LAD)-induced MI mouse model was employed and treated with *Astragalus propinquus* Schischkin and *Salvia miltiorrhiza* Bunge. The purpose of this study was to explore the effect of the above drug pair on protecting cardiac function and angiogenesis after MI, and to elucidate the relationship with pericyte recruitment in a targeted manner.

## 2 Materials and methods

### 2.1 Preparation of AS

*Astragalus propinquus* Schischkin (HuangQi in Chinese pharmacopoeia, family *Leguminosae*) granules and *Salvia miltiorrhiza* Bunge (DanShen in Chinese pharmacopoeia, family *Lamiaceae*) granules were purchased from Beijing Kangrentang Pharmaceutical Co., Ltd. (Beijing, China). According to the manufacturing standard of Chinese formula granules, 2500 g *Astragalus propinquus* Schischkin was boiled with water, filtered and concentrated into clear extract (the extraction rate of dry extract ranged from 22% to 40%). An appropriate amount of excipients was added. These extracts were dried (or dried, crushed), mixed, granulated, and made into 1000 g. 2000 g *Salvia miltiorrhiza* Bunge was boiled with water, filtered and concentrated into clear extract (the extraction rate of dry extract ranged from 31% to 49%). An appropriate amount of excipients was added. These extracts were dried (or dried, crushed), mixed, granulated, and made into 1000 g. Therefore, 1 g of *Astragalus propinquus* Schischkin formula granules are equivalent to 2.5 g of the botanical drugs, and 1 g of *Salvia miltiorrhiza* Bunge formula granules are equivalent to 2 g of the botanical drugs. According to the drug instructions and Chinese Pharmacopoeia, the chemical profiles of *Astragalus propinquus* Schischkin and *Salvia miltiorrhiza* Bunge were attached to the supplementary materials. The above granules were dissolved in .9% NaCl and stored at 4° in the dark.

### 2.2 Animals

7-week-old male C57BL/6J normal and MI mice were purchased from Vital River Laboratory Animal Technology Co. (Beijing, China) and used in the following experiments. The animal experiments were performed in accordance with the Guide for the Care and Use of Laboratory Animals (published by the US National Institutes of Health) and were approved by the Institutional Animal Care and Research Advisory Committee of the Shandong University of Traditional Chinese Medicine. All mice were housed under SPF laboratory conditions with water and food temperature of 22°C ± 2°C and maintained a 12-h light/dark cycle throughout the experiment. The above mice were divided into 3 groups, (n = 8 rats per group), as follows: 1) Control group: Normal mice received LAD sham operation and were gavaged with .9% NaCl (2 mL kg^−1^); 2) Model group: MI mice were gavaged with .9% NaCl (2 mL kg^−1^); 3) AS group: MI mice were gavaged with *Astragalus propinquus* Schischkin and *Salvia miltiorrhiza* Bunge decoction extract (2 mL∙kg-1), which is equivalent to 1.875 g/kg of *Astragalus propinquus* Schischkin and .9375 g/kg of *Salvia miltiorrhiza* Bunge. After 6 weeks of intragastric administration, 3 groups of mice were used for subsequent experiments.

### 2.3 ECG and ultrasonic cardiogram

Mice were anesthetized by inhalation of isoflurane at an initial infusion concentration of 2%, increased to 5% after 3 min, induction of anesthesia was completed within 5 min, and maintained at a concentration of 1% thereafter. The limbs and head of mice were fixed on a wooden mouse board in a supine position, and then four small metal syringe needles were inserted subcutaneously into the limbs. The ECG changes in the limbs were recorded with RM6240E multichannel physiological signal acquisition system (Shanghai, Xinruan, China), recorded from lead I. M-mode images of the left ventricle were obtained by a small animal ultrasound instrument (Xuzhou China) to determine percentage ejection fraction (EF), short axis shortening of the left heart asphyxia (FS), left ventricular end diastolic volume (LVvol; d), left ventricular end systolic volume (LVvol; s), left asphyxiating end diastolic diameter (LVID; d) and left ventricular end systolic diameter (LVID; s).

### 2.4 TTC staining

The mouse heart tissues were frozen at—80°C for 10 min, and then cut into five 2 mm thick slices along the transverse axis. The heart slices were immersed in 1% TTC solution and dyed at 37°C for half an hour. Next, the hearts were placed in order and photographed. The normal heart tissues were red, and the infarcted areas were grayish white. ImageJ software was used to calculate the area of myocardial infarction.

### 2.5 H&E staining

Mouse heart tissue was stored at −20°C for 30 min and cut into 2 mm thick sections, which were then stained with H&E. The staining results were observed under a microscope to detect ischemic myocardium.

### 2.6 Masson staining

Mouse heart tissue was fixed with 3% glutaraldehyde fixative (pH 7.2–7.4) for 24 h, and the aortic sinus was cut into 5 µm serial paraffin sections. Masson (Nanjing Jiancheng Technology Co., Ltd., Nanjing, China) was performed to determine collagen fiber content. The staining results were observed under a fluorescence microscope for pathological analysis and analysed semi-quantitatively based on the collagen volume fraction (CVF, percentage of collagen-positive blue area to total tissue area) using ImageJ software.

### 2.7 Immunohistochemical (IHC) staining

Mouse heart tissues were fixed in 4% paraformaldehyde and embedded in paraffin to cut into 5 μm sections. The sections were blocked in blocking buffer at room temperature and then incubated with CD31: (1:100, ab24590, Abcam), PDGFR-β: (1:100, #3169, Cell Signaling) at 4° for 12 h according to the instructions. After washing the sample sections, the sections were incubated with the secondary antibody for 1.5 h at room temperature. The staining results were observed under a microscope to explore the recruitment of pericytes around endothelial cells.

### 2.8 Immunofluorescence (IF) staining

The mouse heart sections were first treated with EDTA Antigen Retrieval Solution (C1033, Solarbio) according to the instructions, and incubated with .5% Triton X-100 for 30 min. After being blocked with 3% BSA for 30 min, the mouse heart slices were incubated with FAK (1:500, 66258-1-lg, Proteintech) at 4°C overnight. CoraLite488 combined Goat Anti Mouse IgG (H + L) (1:500, SA00013-1, Proteintech) was used as a secondary antibody to incubate the slices in dark at room temperature for 50 min. The slices were dropped with DAPI containing anti fluorescence attenuation sealing agent (S2110, Solarbio), and visualized using a fluorescent microscope.

### 2.9 Enzyme-linked immunosorbent assay (ELISA)

Blood samples were collected and left to stand for 30 min, then were centrifuge for 15 min at 3000r at 4°C. BNP, NT-ProBNP, Ang-1, Ang-2 and Tie-2 in the supernatant was measured using the Mouse ELISA Kit (Elabscience, Wuhan, China). The absorbance was recorded at 450 nm with a plate reader. Based on the standard curve OB values of the samples, the corresponding BNP, NT-ProBNP, Ang-1, Ang-2 and Tie-2 were calculated.

### 2.10 Western blotting

The mice myocardial tissues were lysed with RIPA buffer (R0020, Solarbio), with protease inhibitor (.1% phenylmethanesulfonyl fluoride (PMSF) (P0100, Solarbio)). Protein concentrations were measured using a BCA protein assay kit (Interchim, Montluçon, France). The proteins were separated by 10% SDS-polyacrylamide gel electrophoresis (SDS-PAGE), and the resulting protein bands were transferred onto PVDF membranes. After blocking with 5% fat-free dry milk in Tris-buffered saline containing .1% Tween-20 for 2 h, the membranes were incubated with the following primary antibodies overnight at 4°C: Collagen 1 (1:1000, #72026, Cell Signaling), matrix metalloproteinase-9 (MMP9) (1:1000, 10375-2-AP, Proteintech), PDGFR-β (1:1000, 13449-1-ap, Proteintech), NG2 (1:1000, #4226, Cell Signaling), VE-cadherin (1:1000, ab33168, Abcam), VEGF (1:1000, 19003-1-ap, Proteintech), TGF-β (1:1000, 21898-1-ap, Proteintech), Ang-1 (1:1000, ab183701, Abcam), Ang-2 (1:1000, ab155106, Abcam), phospho-Tie-2 (1:1000, #4226, Cell Signaling), GRB7 (1:1000, 10045-1-Ig, Proteintech), FAK (1:1000, ab40794, Abcam) and β-actin (1:1000, 66009-1-lg, Proteintech). Then, the membranes were incubated with horseradish peroxidase-conjugated secondary antibodies at room temperature for 1 h. The protein bands were visualized using an ECL Substrate (Thermo Fisher Scientific), and the quantification of the average densities of the protein bands was performed using the ImageJ software.

### 2.11 RNA isolation and quantitative-PCR (q-PCR)

The total RNA in mouse heart tissues was extracted with TriZol. After the concentration was determined, the RNA was reverse transcribed into cDNA using SPARKscript II RT Plus Kit (AG0304, SparkJade). SYBR Green qPCR Mix (Ah0104, SparkJade) was used to amplify the target gene, and the reaction system was 10 μL. Roche LightCycle 480II was used for qPCR experiments. The primer sequences for Collagen 1, MMP9 and β-actin were listed in Table 1.

**TABLE 1:** The primer sequences for Collagen 1, MMP9 and β-actin.

Gene of interest	Species	Sequence (5′-3′)
Collagen 1	Mouse	Forward: CCC​TGG​TCC​CTC​TGG​AAA​TG
		Reverse: GGA​CCT​TTG​CCC​CCT​TCT​TT
MMP9	Mouse	Forward: TAG​ATC​ATT​CCA​GCG​TGC​CG
		Reverse: GCT​TAG​AGC​CAC​GAC​CAT​ACA
β-actin	Mouse	Forward: GGC​TGT​ATT​CCC​CTC​CAT​CG
		Reverse: CCA​GTT​GGT​AAC​AAT​GCC​ATG​T

### 2.12 Statistical analysis

Data from at least three independent experiments were analyzed using Graphpad 7 software (GraphPad Software, La Jolla, CA, United States) and are expressed as the mean ± standard deviation (SD). Two groups of data were compared using an independent samples *t*-test, while multiple groups were compared using one-way ANOVA with Bonferroni’s multiple comparison *post hoc* test. *p*-values <.05 were considered statistically significant.

## 3 Results

### 3.1 AS reduces MI and protects cardiac function

At baseline, there was no significant difference in ECG between model group and AS group, whereas ST segment of the two groups was higher than that of the control group, indicating that the MI mouse model was successfully established.

**FIGURE 1:**
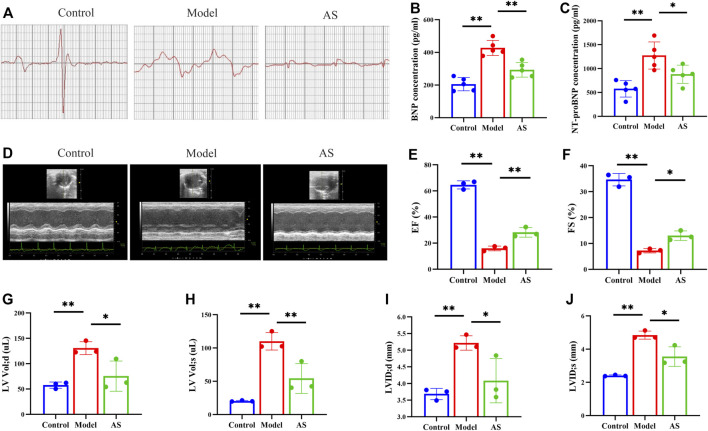
AS reduces MI and protects cardiac function. **(A)** Representative images of Mice electrocardiogram. **(B)** Quantitative analysis of BNP concentration, n = 5. **(C)** Quantitative analysis of NT-proBNP concentration, n = 5. **(D)** Representative images of mice echocardiography. **(E)** Quantitative analysis of EF (%), n = 3. **(F)** Quantitative analysis of FS (%), n = 3. **(G)** Quantitative analysis of LVVd; d (uL), n = 3. **(H)** Quantitative analysis of LVVd; s (uL), n = 3. **(I)** Quantitative analysis of LVID; d (mm), n = 3. **(J)** LVID; s (mm), n = 3. Results are expressed as mean ± SD, **p < .001, *p < .05.

In clinical practice, electrocardiography has been used for more than a century to detect MI and drug effects (Yan et al., 2003). Therefore, we first carried out mouse ECG detection. After 6 weeks of intervention, the ECG of the model group showed the most significant ST segment shift (i.e., the magnitude of distortion) and the longest time interval for repolarization abnormalities (i.e., ST segment integration). (Figure 1A). These results suggest that MI affects cellular depolarization and repolarization processes, resulting in decreased cardiomyocyte resting potential, production of pathological ionic currents, and opening of ATP-K channels, thereby affecting T wave morphology and duration (Yan et al., 2003). In the AS group, these ischemia indicators were partially restored, suggesting that the botanical drugs could alleviate the damage caused by MI to a certain extent.

Then, echocardiography in short-axis M-mode mode was performed (Figure 1D). The results showed that there was improvement in EF, FS (Figures 1E, F) and significant reductions in LVVol; d, LVVol; s, LVID; d, LVID; s (Figures 1G–J) in AS mice compared to the model.

Since cardiac function was preserved after AS intervention in MI mice, the next goal was to determine whether AS could slow disease progression and delay the onset of heart failure. To accomplish this, two markers of heart failure, BNP and NT-ProBNP, were measured by ELISA. The serum expression levels of these two indicators are of great significance, not only reflecting cardiovascular defects, but also their severity (Maries and Manitiu, 2013). Previous studies have confirmed that the levels of BNP and NT-proBNP are positively correlated with the risk of death, which can effectively evaluate the prognosis and drug treatment effects of MI mice (Nunez et al., 2008). As shown in Figures 1B, C, the levels of BNP and NT-ProBNP in control group were maintained at a low level, while those in model group were significantly increased due to ischemia. At the same time, the expressions of BNP and NT-ProBNP in AS group were lower than those in model group, indicating that the botanical drugs played a protective role.

### 3.2 AS attenuates myocardial pathological injury

**FIGURE 2:**
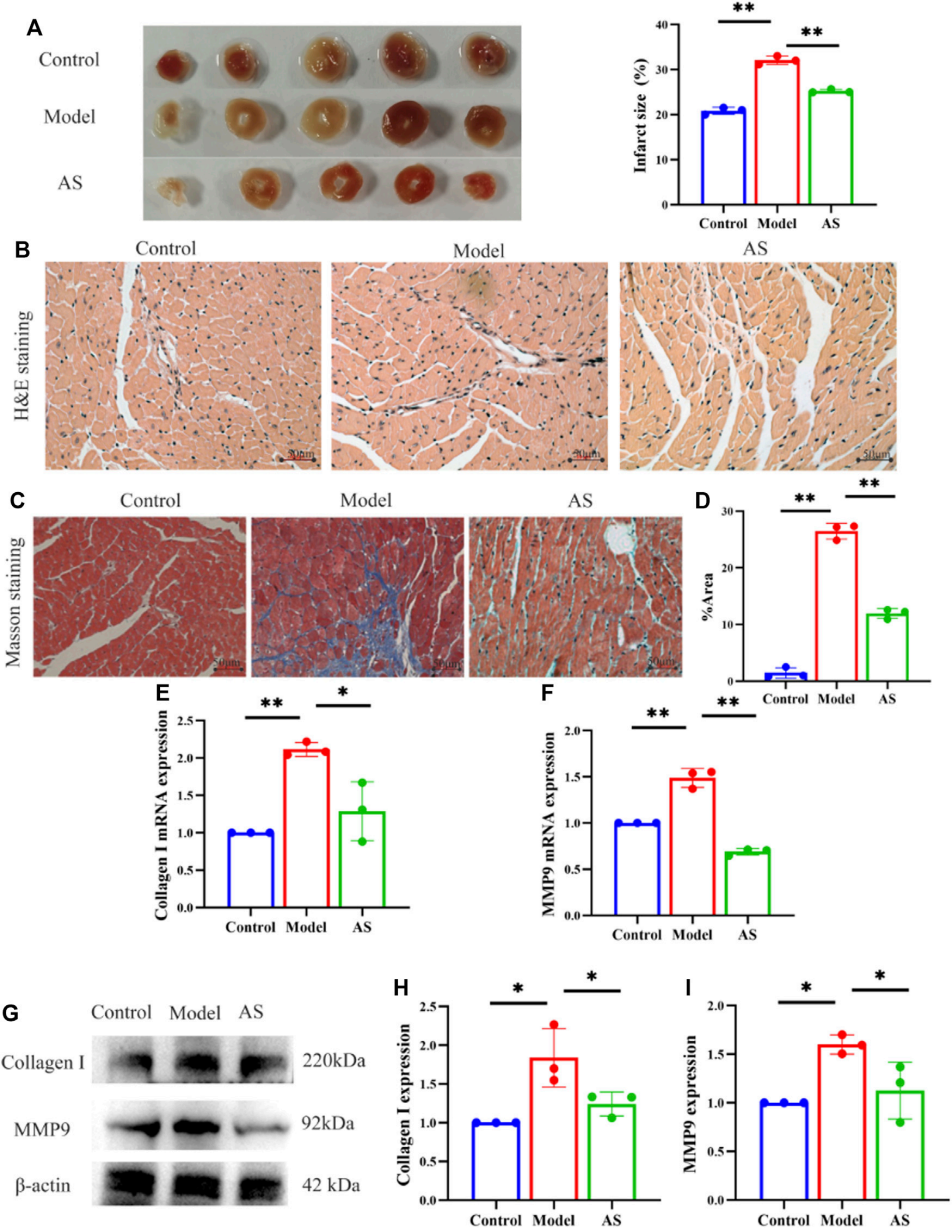
AS attenuates myocardial pathological injury. **(A)** Representative images and calculated infarct size of the myocardium of mice. **(B)** Representative images of H&E staining. **(C)** Representative images of Masson staining. **(D)** Fibrosis area as percentage, results are expressed as mean ± SD, n = 3, **p < .01. **(E)** Quantitative analysis of Collagen I mRNA, results are expressed as mean ± SD, n = 3, **p < .01, *p < .05. **(F)** Quantitative analysis of MMP9 mRNA, results are expressed as mean ± SD, n = 3, **p < .01. **(G)** Western blot analysis of Collagen I and MMP9 expression in mice. **(H)** Quantitative analysis of Collagen I, results are expressed as mean ± SD, n = 3, *p < .05. **(I)** Quantitative analysis of MMP9, results are expressed as mean ± SD, n = 3, *p < .05.

To investigate the mitigating effect of AS on cardiac pathological changes after MI, histological staining was performed. TTC staining showed that after LAD operation, the MI area of mice increased to 31%, while after AS intervention, the index decreased to 25%. (Figure 2A).

On this basis, we observed the pathological damage of MI in mice from a more microscopic level. After HE staining of myocardial tissue under light microscope, it was found that the structure of blood vessels in the control group was normal, and the myocardial fibers were arranged neatly. In the model group, the arrangement of myocardial fibers was disordered, the vascular wall was edema, and inflammatory cell infiltration was seen around the blood vessels. Compared with the model group, the myocardial fibers in the AS group were still neatly arranged, and the vascular wall edema and inflammatory cell infiltration were reduced (Figure 2B).

In addition, Masson staining showed that the cardiomyocytes in control group were red-stained, with normal size, neat arrangement, clear structure, and almost no blue-stained collagen fibers. In the model group, the structure of myocardial cells was disordered, with extensive blue staining of collagen fibers, which was more obvious near blood vessels. And cardiomyocytes were divided into strips or islands by collagen fibers. AS treatment reduced myocardial fiber rupture and collagen deposition, resulting in a lower proportion of myocardial fibrosis than model group (Figures 2C, D).

In order to further examine the antagonistic effect of AS on myocardial fibrosis after MI, collagen 1 and MMP9 were measured from the RNA and protein layers. Collagen 1 is considered to be the decisive factor leading to myocardial fibrosis, and MMP9 has also been proved to be an important reason for extracellular matrix remodeling after MI, which are closely related to left ventricular remodeling (Garcia et al., 2005; Wang et al., 2014). Consistent with the results of cardiac staining, AS intervention can reduce the expression of collagen 1 and MMP9 induced by MI to some extent. These indicate that AS can reduce myocardial pathological damage after MI by inhibiting myocardial fibrosis (Figures 2E–I).

### 3.3 AS regulates pericyte recruitment and promotes angiogenesis

**FIGURE 3:**
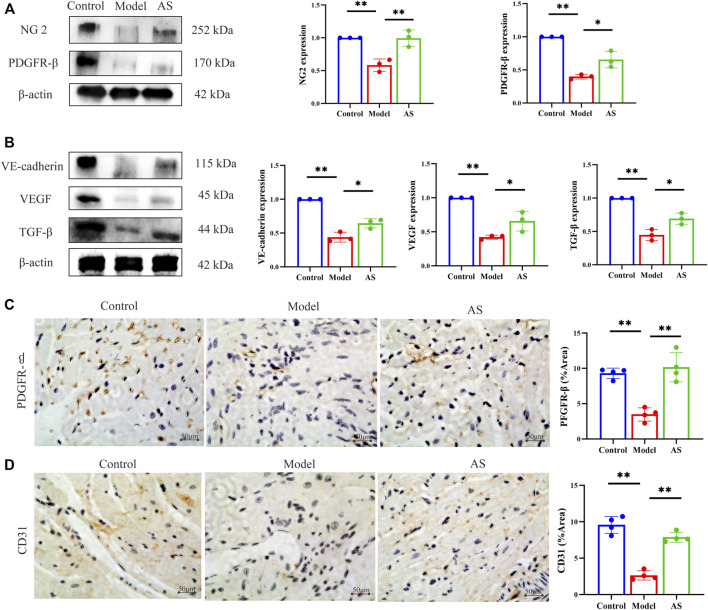
AS activates pericyte recruitment and promotes angiogenesis in MI mice. **(A)** Protein level of NG 2 and PDGFR-β, results are expressed as mean ± SD, n = 3, **p < .01, *p < .05. **(B)** Protein level of VE-cadherin, VEGF and TGF-β, results are expressed as mean ± SD, n = 3, **p < .01, *p < .05. **(C)** IHC staining, the brownish-yellow region is PDGFR-β, results are expressed as mean ± SD, n = 4, **p < .01. **(D)** IHC staining, the brownish-yellow region is CD31, results are expressed as mean ± SD, n = 4, **p < .01.

Whether AS can treat MI by promoting angiogenesis through pericyte recruitment? We further confirmed this by measuring the expression of NG2 and PDGFR-β, which are markers of pericytes. NG2 is expressed in microvascular pericytes in neovascularization and can mediate the communication between pericytes and endothelial cells. Therefore, NG2 is an important factor to promote endothelial cell migration and morphogenesis in the early stage of neovascularization (Fukushi et al., 2004). PDGF receptor signaling pathway plays a key role in pericytes recruitment during angiogenesis (Lindahl et al., 1997). WB results showed that in the model group, the expression of NG2 and PDGFR-β decreased. In contrast, the expression increased after AS treatment, (Figure 3A), which indicate that pericytes are recruited near endothelial cells for proliferation and migration.

After pericytes are collected around endothelial cells, they secrete VEGF to act on endothelial cells and promote the stability and maturation of new blood vessels. Pericytes and endothelial cells can also co-secrete TGF- β. In addition, VE-cadherin can maintain the adhesion between cells and the integrity of new blood vessels. As shown in Figure 3B, AS can mitigate the decline of VEGF, TGF-β and VE-cadherin caused by MI.

We further provided solid evidence through IHC. The cells stained with brownish yellow containing PDGFR-β and CD31 decreased significantly in model group, and recovered in AS group (Figures 3C, D). These evidences indicate that AS can treat MI by promoting angiogenesis through pericyte recruitment.

### 3.4 AS regulates Ang-1/Tie-2/FAk pathway

**FIGURE 4:**
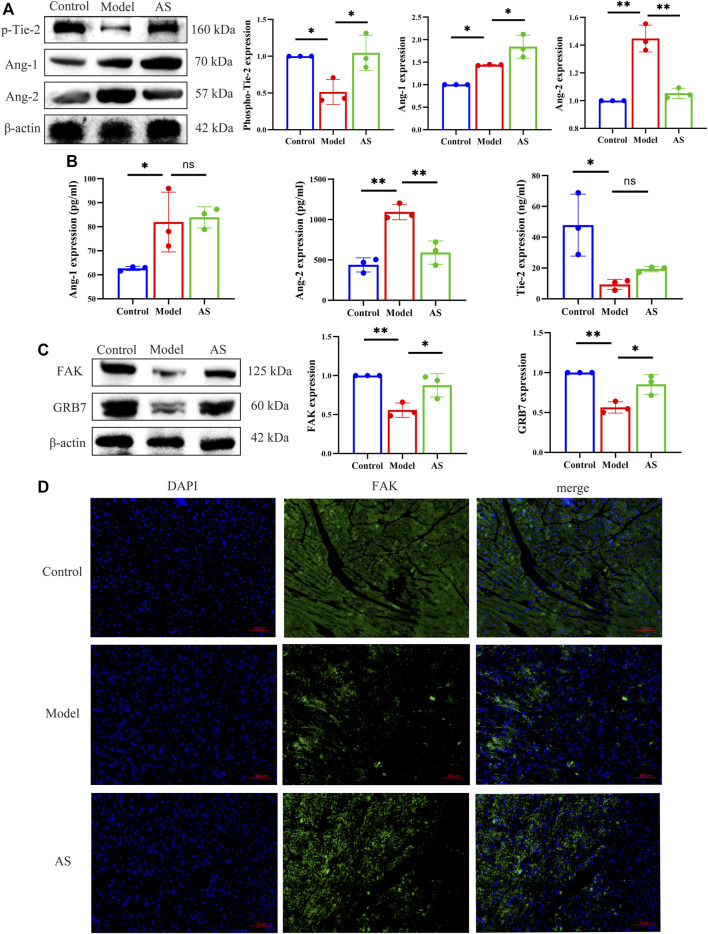
AS regulates Ang-1/Tie-2/FAk pathway. **(A)** Protein level of p-Tie-2, Ang-1 and Ang-2, results are expressed as mean ± SD, n = 3, **p < .01, *p < .05. **(B)** Quantitative analysis of Ang-1, Ang-2 and p-Tie-2 concentration, n = 3. Results are expressed as mean ± SD, **p < .001, *p < .05, ns indicates not significant. **(C)** Protein level of FAK and GRB7, results are expressed as mean ± SD, n = 3, **p < .01, *p < .05. **(D)** IF staining, the green region is FAK, results are expressed as mean ± SD, n = 3, **p < .01, *p < .05.

Since AS can promote pericyte recruitment and angiogenesis, the next goal is to determine how AS can promote angiogenesis in the heart after MI. Thus, classical angiogenic proteins, Ang-1, Ang-2 and p-Tie-2 were detected. As shown in Figure 4A, the expression of p-Tie-2 in model group was lower than that in normal group, while that in AS group was higher than that in model group. In contrast to p-Tie-2, Ang-2 was most expressed in the model group. Compared with the model group, the expression of Ang-2 in AS group was lower. Notably, the expression of Ang-1 increased after MI, and the intervention of AS can enhance this rising trend. We further explored by ELISA and got the same trend as the WB results above (Figure 4B).

In order to investigate the downstream pathway of Ang-1/Tie-2, we explored the expression of GRB7 and FAK in mice. Compared with LAD sham operated mice, the levels of GRB7 and FAK in model group mice decreased significantly. Treatment of MI mice with AS increased the production of GRB7 and FAK (Figure 4C). And in Figures 4D, I, F showed the same results.

## 4 Discussion

Angiogenesis is a new therapeutic direction for MI, and we propose that two botanical drugs, *Astragalus propinquus* Schischkin and *Salvia miltiorrhiza* Bunge, can promote angiogenesis through pericyte recruitment. Therefore, we took the sham-operated mice as control group, the LAD mice as model group, and the LAD mice gavaged with extracts of *Astragalus propinquus* Schischkin and *Salvia miltiorrhiza* Bunge as AS group. The first objective of this study was to determine the extent of cardioprotection after MI by AS. The results of ECG and echocardiography showed that compared with the model group, the ST segment injury degree and abnormal repolarization time in the AS group were reduced, and the EF, FS, LVVol; d, LVVol; s, LVID; d, LVID; s were recovered. Next, we performed an ELISA to test for BNP and NT-proBNP, and the lower values compared to the model group indicated that AS intervention could slow the progression of heart failure. Further cardiac pathology analysis demonstrated a smaller infarct size, a more stable cardiac structure and a reduced collagen deposition in AS group, when compared to model group. Further research found that the pericyte-specific marker PDGFR-β, NG2 and endothelial cell-specific marker CD31 in AS group rebounded. In addition, the angiogenesis related proteins VE cadherin, VEGF and TGF- β also increased after AS intervention compared with the model group, indicating that the effects of AS in the treatment of MI were related to pericyte recruitment and angiogenesis. Finally, Western blotting and IF results showed that AS played the role as described above through Ang-1/Tie-2/FAK pathway.

Interestingly, the results showed that AS upregulated the expression of Ang-1 and downregulated the expression of Ang-2. Ang-1 and Ang-2, which are secreted proteins that bind Tie-2 receptors, have the ability to regulate vascular maturation (Dumont et al., 1992; Korhonen et al., 1992). Ang-1 mainly promotes vascular maturation and maintains homeostasis, while Ang-2 can break vascular homeostasis and allow angiogenesis or regression. (Maisonpierre et al., 1997; Kim et al., 2000a). Previous studies have shown that Ang-1 overexpression can normalize the immature vascular system and increase the generation of new blood vessels, which is accompanied by a significant improvement in the density of capillaries and arterioles induced by ischemia in the heart infarction region (Chen and Stinnett, 2008). Therefore, we conclude that after MI, the stress response caused by ischemia and hypoxia increased the expression of Ang-1 in mice, and the regulatory mechanism of the heart tried to antagonize MI damage through spontaneous angiogenesis. In this case, AS expands this trend of angiogenesis and further increased the expression of Ang-1. In addition, Ang-2 is also highly expressed in MI mice. Studies have shown that Ang-2 begin to express on the second day after MI, reaches the peak on the third day, and remains at the peak level until the seventh day (Lee et al., 2018). This may be related to Ang-2’s ability to start angiogenesis, because in the case of high concentration of Ang-2, it can be used as an agonist of angiogenesis (Kim et al., 2000b; Wakui et al., 2006). It is worth noting that during acute MI, Ang-1 and Ang-2 exist at the same time, which will cause Ang-2 to antagonize Ang-1/Tie-2 signal and aggravate cardiac hypoxia (Lee et al., 2018). As a consequence, we speculate that after MI, the expression of Ang-2 increases to start the process of angiogenesis, and the application of AS weakens the expression of Ang-2, thereby weakening its negative effect against the maturation and stability of neovascularization.

Furthermore, as an antigen of Tie2, GRB7 can activate FAK to promote angiogenesis. FAK, a cycloplasmic tyrosine kinas, is a downstream protein of Ang-1 and Tie-2. Previous studies have shown that the interaction between Tie-2 and FAK is related to cell migration (Lamalice et al., 2007). Under the control of Tie-2 promoter and enhancer, FAK can promote angiogenesis in ischemic model (Peng et al., 2004). At the same time, Ang-1 can promote the phosphorylation of FAK (Kim et al., 2000c). In addition, the interaction between FAK and GRB7 is also related to stress response, which may also affect angiogenesis (Tsai et al., 2008).

We further focused on the active ingredients of AS. Previous studies have shown that *Astragalus propinquus* Schischkin and its main active components astragaloside IV and astragalus polysaccharide can exert cardioprotective effects *in vitro* and *in vivo*, which are specifically manifested in inhibiting myocardial cell death, reducing oxidative stress, reducing autophagosome accumulation, reducing myocardial fibrosis. and myocardial remodeling, thereby improving cardiac function and reducing cardiac ischemia-reperfusion injury (Ma et al., 2013; Liu et al., 2018; Jiang et al., 2019; Huang et al., 2021; Zhang et al., 2022). *Salvia miltiorrhiza* Bunge, with tanshinone IIa, salvianolic acid A and B as the main active ingredients, has also been proven to prevent and treat MI, cardiac hypertrophy and cardiac fibrosis (Li et al., 2018). Specifically, *Salvia miltiorrhiza* Bunge extract can regulate the accumulation of free fatty acids in ischemic myocardium (Sun et al., 2021), inhibit the expression of endothelial cell adhesion molecules (Jin et al., 2009), and improve ischemia-reperfusion microcirculation disorders and target organ damage (Han et al., 2008). Furthermore, the combination of *Astragalus propinquus* Schischkin and *Salvia miltiorrhiza* Bunge widely used in China and can enhance the effect on MI.

**FIGURE 5:**
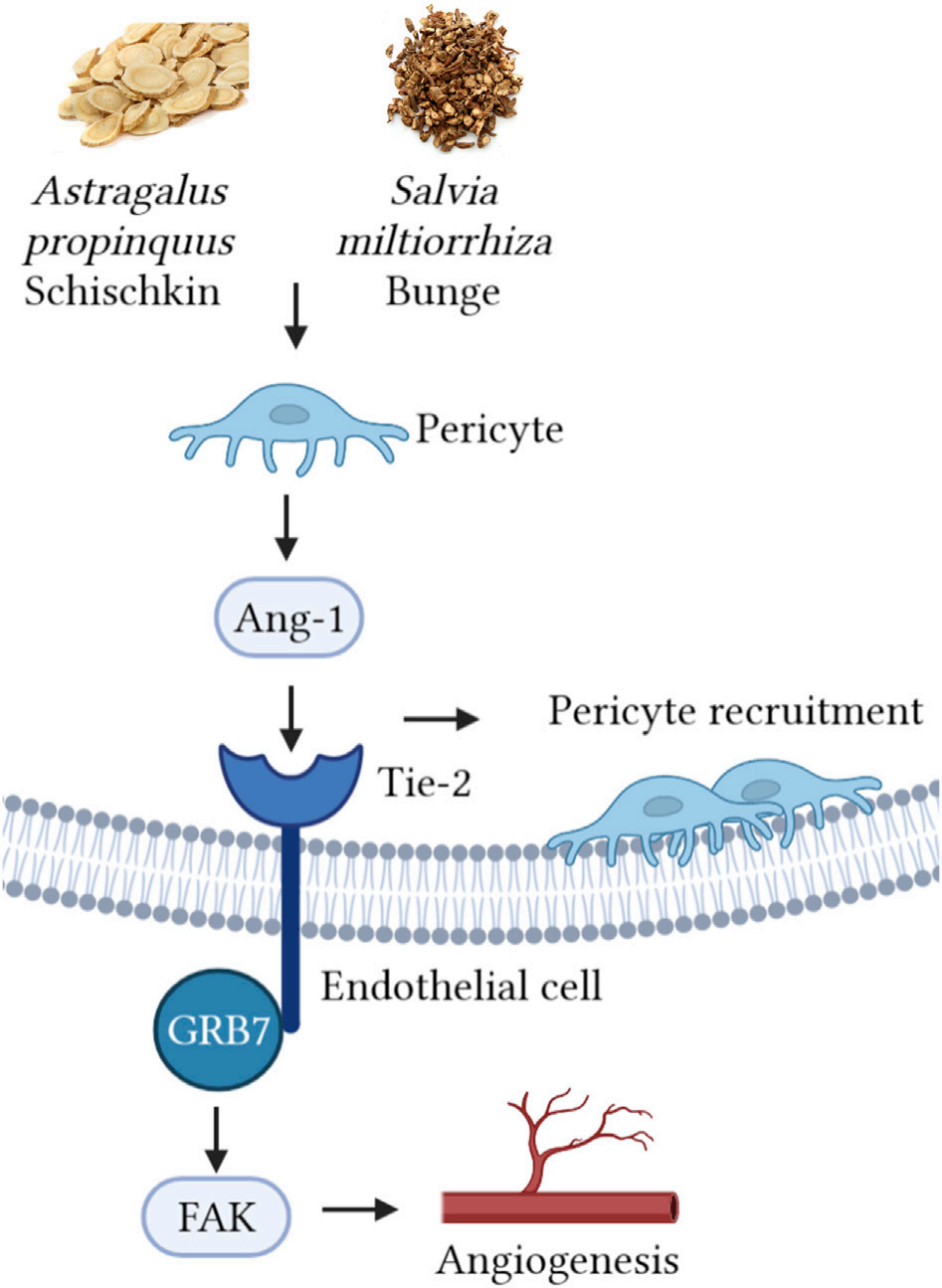
*Astragalus propinquus* Schischkin and *Salvia miltiorrhiza* Bunge regulate pericyte recruitment and promote angiogenesis to treat MI *via* Ang-1/Tie-2/FAk pathway.

In this study, we focused on the synergistic ability of AS, explored their effect on promoting angiogenesis after MI, and innovatively pointed out the relationship between botanical drugs and pericyte recruitment. As shown in Figure 5, we speculate that this therapeutic effect is related to the regulation of the pericyte Ang-1/Tie-2/FAK paracrine loop by the botanical drugs. However, this study has some limitations. In the *in vivo* experiment, we only used the single dose recommended by the drug manual, which conforms to the specifications of Chinese Pharmacopoeia, but the concentration gradient was not set. This is an early exploration, and we aim to explore the effect of AS on promoting angiogenesis after MI and its possible mechanism. Based on the 4R rules (Reduce, refine, replace—Responsibility), we simplified the experimental procedures in order to reduce the pain or discomfort caused to animals, reduce the frequency and harm of inhuman use, and improve animal welfare. In the follow-up study, we will explore the relationship between dose and efficacy, screen out the main effective components of *Astragalus propinquus* Schischkin and *Salvia miltiorrhiza* Bunge, and further explore their mechanisms through *in vivo* and *in vitro* experiments. We expect that the effect of *Astragalus propinquus* Schischkin and *Salvia miltiorrhiza* Bunge on promoting angiogenesis can be applied to clinical practice and provide new complementary and alternative therapies for patients with MI.

## 5 Conclusion

Taken together, we demonstrate the efficacy of AS on MI at a single dose. AS has a positive effect on protecting cardiac function, hindering myocardial pathological changes, and slowing down the process of heart failure, which is achieved by regulating the recruitment of pericytes, thereby promoting angiogenesis. Our findings provide new complementary and alternative options for the future treatment of MI.

## Data Availability

The raw data supporting the conclusion of this article will be made available by the authors, without undue reservation.

## Glossary

Ang-1: Angiopoietin-1
Ang-2: Angiopoietin-2
AS: *Astragalus propinquus* Schischkin and Salvia miltiorrhiza Bunge
BNP: Brain Natriuretic Peptide
CABG: coronary artery bypass graft
CD31: platelet endothelial cell adhesion molecule-1
ECG: Electrocardiogram
EF: ejection fraction
ELISA: Enzyme-linked Immunosorbent Assay
FAK: focal adhesion kinase
FS: short axis shortening of the left heart asphyxia
GRB7: growth factor receptor bound protein 7
H&E: Hematoxylin and eosin
IF: immunofluorescence
IHC: immunohistochemistry
LAD: left anterior descending coronary artery ligation
LVID; d: left asphyxiating end diastolic diameter
LVID; s: left ventricular end systolic diameter
LVvol; d: left ventricular end diastolic volume
LVvol; s: left ventricular end systolic volume
MI: myocardial ischemia
MMP9: matrix metalloproteinase-9
NT-proBNP: N-terminal Pro-brain Natriuretic Peptide
PCI: percutaneous coronary intervention
PDGFR-β: platelet-derived growth factor receptors β
p-Tie-2: phosphorylated angiopoietin-1 receptor
TGF-β: transforming growth factor β
q-PCR: RNA isolation and quantitative-PCR
SD: standard deviation
TTC: Triphenyl tetrazolium chloride
VE-cadherin: vascular endothelial cadherin
VEGF: Vascular Endothelial Growth Factor
WHO: World Health Organization
