# Supplementary material for: Astragalus propinquus schischkin and Salvia miltiorrhiza bunge promote angiogenesis to treat myocardial ischemia via Ang-1/Tie-2/FAK pathway
Source: Front Pharmacol. 2023 Jan 9;13:1103557. doi: 10.3389/fphar.2022.1103557 (PMC9868545; doi:10.3389/fphar.2022.1103557)
Supplement: Supplementary file 1 [file DataSheet1.docx]

Supplementary Material

*Astragalus propinquus* Schischkin and *Salvia miltiorrhiza* Bunge promote angiogenesis to treat myocardial ischemia via Ang-1/Tie-2/FAK pathway

Mu-xin Zhang^1†^, Xue-ying Huang^2†^, Yu Song^3^, Wan-li Xu^4^, Yun-lun Li^3,5^, Chao Li^3*^

^1^ First Clinical Medical College, Shandong University of Traditional Chinese Medicine, Jinan, China

^2^ College of Pharmacy, Shandong University of Traditional Chinese Medicine, Jinan, China

^3^ Innovation Research Institute of traditional Chinese Medicine, Shandong University of Traditional Chinese Medicine, Jinan, China

^4^ College of Traditional Chinese Medicine, Shandong University of Traditional Chinese Medicine, Jinan, China

^5^ Department of Cardiology, The Affiliated Hospital of Shandong University of Traditional Chinese Medicine, Jinan, China

*** Correspondence:**Chao Li
lichao71795@hotmail.com

Mu-xin Zhang†, Xue-ying Huang† These authors contributed equally to this work and share first authorship

# Chemical profile of *Astragalus propinquus* Schischkin

## Extraction method

2500g *Astragalus propinquus* Schischkin was boiled with water, filtered and concentrated into clear extract (the extraction rate of dry extract ranged from 22% to 40%). An appropriate amount of excipients was added. These extracts were dried (or dried, crushed), mixed, granulated, and made into 1000g.

## Chromatographic conditions and system suitability test

Octadecylsilane bonded silica gel was used as filler, acetonitrile was used as mobile phase A, and 0.02% formic acid solution was used as mobile phase B. The gradient elution method was recorded in Supplementary Table 1. The flow rate was set to 1ml per minute and the column temperature was 30 ℃. The ultraviolet detector and evaporative light scattering detector were used for detection respectively, and the detection wavelength of the ultraviolet detector was 230 nm. The number of theoretical plates shall not be less than 3000 according to the peak of pistil isoflavone glucoside.

| Time (minutes) | Mobile phase A (%) | Mobile phase B (%) |
| --- | --- | --- |
| 0~30 | 20→45 | 80→55 |
| 30~60 | 45→80 | 55→20 |

**Supplementary Table 1.** Gradient elution method in the preparation of *Astragalus propinquus* Schischkin extract.

## Preparation of reference solution

1g *Astragalus propinquus* Schischkin control drug was put into a conical flask with a stopper, and 10ml of 30% methanol was added. The conical flask was tightly plugged, heated and refluxed for 30 minutes, cooled, shaken and filtered. The filtrate was used as the reference solution of the control medicine. Then, a proper amount of reference substance of pistil isoflavone, pistil isoflavone glucoside, astragaloside II and astragaloside I was precisely weighed, and methanol was added to prepare a reference substance solution containing 50 µg *Astragalus propinquus* Schischkin control drug per lml.

## Preparation of test solution

An appropriate amount of *Astragalus propinquus* Schischkin was ground, and 1g of it was prepared into the test solution according to the preparation method in Section 2.1.

## Determination method

The reference solution and the test solution were precisely aspirated by 10 µ l respectively, injected into the liquid chromatograph, and measured.

## Chromatogram of test sample (ultraviolet detection)

The chromatogram of the test sample (ultraviolet detection) showed 4 characteristic peaks, which corresponded to the retention time of the 4 characteristic peaks in the chromatogram of the reference drug. Among them, peak 2 and peak 3 correspond to the retention time of pistil isoflavone glucoside and pistil isoflavone respectively. The peak corresponding to the reference substance peak of pistil isoflavones was the S peak. Calculate the relative retention time of each characteristic peak and S peak, and the relative retention time was within ± 10% of the specified value. The specified values were 0.18 (peak 1) and 1.58 (peak 4). (Supplementary Figure 1)


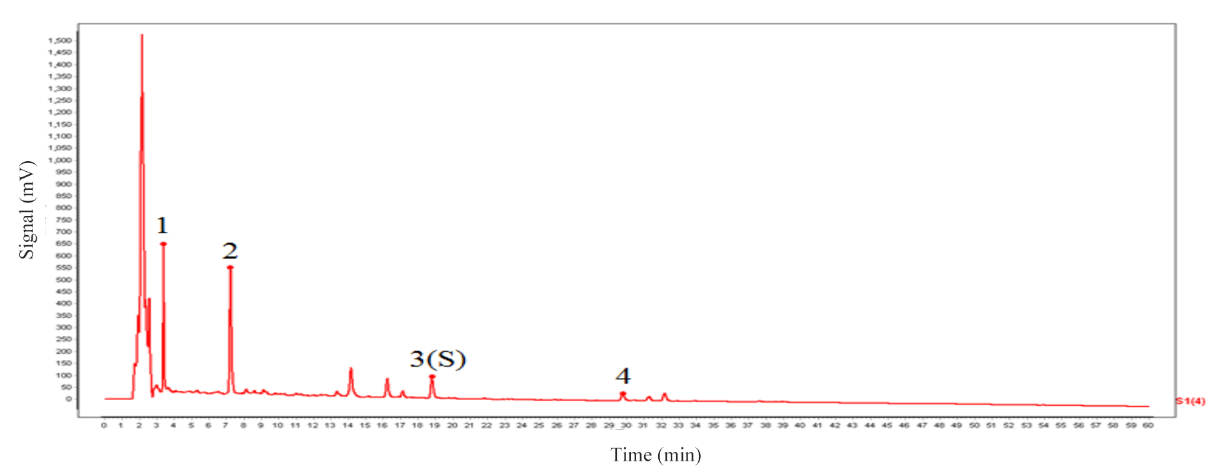


**Supplementary Figure 1.** Contrast characteristics (HPLC-DAD). Peak 2: pistil isoflavone glucoside; Peak 3 (S): pistil isoflavones. Chromatographic column: Lichrosphere C18, 4.6mm × 250mm，5μm.

## Chromatogram of test sample (evaporative light scattering detection)

The chromatogram of the test sample (evaporative light scattering detection) showed 5 characteristic peaks, which corresponded to the retention time of the 5 characteristic peaks in the chromatogram of the reference drug. Among them, peak 1-4 correspond to the retention time of pistil isoflavone glucoside, pistil isoflavone, Astragaloside II and Astragaloside I respectively. The peak corresponding to the reference substance peak of Astragaloside I was the S peak. Calculate the relative retention time of each characteristic peak and peak 5, and the relative retention time was within ± 10% of the specified value. The specified value was 1.05 (peak 5). (Supplementary Figure 2)


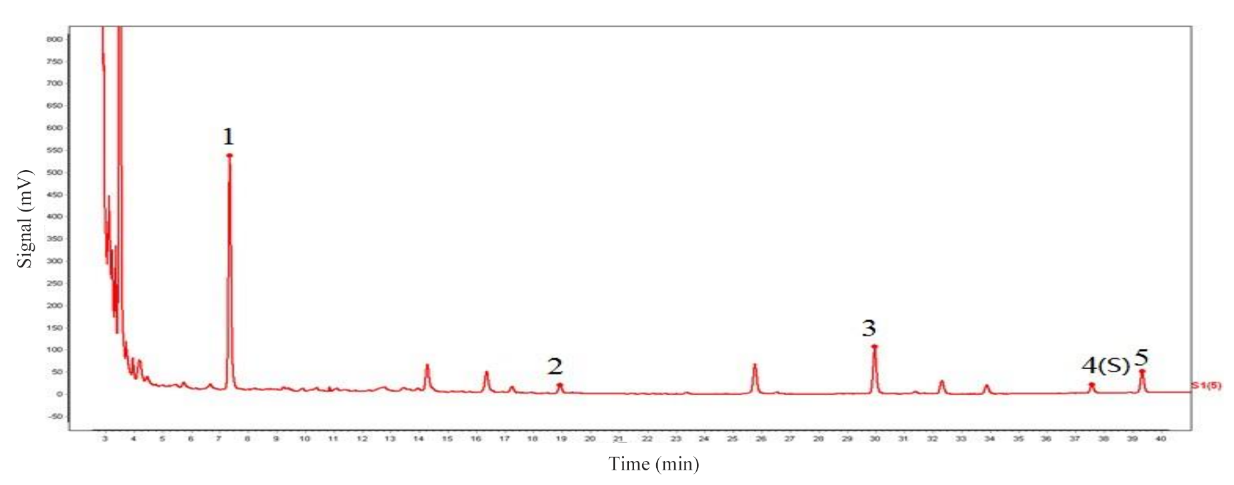


**Supplementary Figure 2.** Contrast characteristics (HPLC-ELSD). Peak 1: pistil isoflavone glucoside. Peak 2: pistil isoflavones. Peak 3: Astragaloside II. Peak 4 (S): Astragaloside I.Chromatographic column: Lichrosphere C18, 4.6mm × 250mm，5μm.

# Chemical profile of *Salvia miltiorrhiza* Bunge

## Extraction method

2000g *Salvia miltiorrhiza* Bunge was boiled with water, filtered and concentrated into clear extract (the extraction rate of dry extract ranged from 31% to 49%). An appropriate amount of excipients was added. These extracts were dried (or dried, crushed), mixed, granulated, and made into 1000g.

## Chromatographic conditions and system suitability test

Octadecylsilane bonded silica gel was used as filler, the length of the column was 25cm, the inner diameter was 4.6mm, and the particle size was 5 µ m. Acetonitrile was used as mobile phase A, and 0.05% phosphoric acid solution was used as mobile phase B. The gradient elution method was recorded in Supplementary Table 2. The detection wavelength was 286 nm.The number of theoretical plates shall not be less than 6000 according to the peak of salvianolic acid B.

| Time (minutes) | Mobile phase A (%) | Mobile phase B (%) |
| --- | --- | --- |
| 0~15 | 10→20 | 90→80 |
| 15~40 | 20→25 | 80→75 |
| 40~50 | 25→30 | 75→70 |

**Supplementary Table 2.** Gradient elution method in the preparation of *Salvia miltiorrhiza* Bunge extract.

## Preparation of reference solution

An appropriate amount of salvianolic acid B was added with methanol-water (8:2) mixed solution to prepare a solution containing 0.10mg salvianolic acid B per 1ml.

## Preparation of test solution

An appropriate amount of *Salvia miltiorrhiza* Bunge was ground, and 0.2g of it was put into a conical flask with a stopper, and 50ml methanol-water (8:2) mixed solution was added. The conical flask was tightly plugged, weighed, ultrasonically treated (power: 140W, frequency: 42kHz) for 30 minutes, taken out, cooled, weighed again, supplemented with methanol-water (8:2)mixed solution to reduce the loss, shaken up, filtered, accurately measured 5ml of continued filtrate into a 10ml volumetric flask, diluted to the scale with methanol-water(8:2) mixed solution, and shaken up.

## Determination method

The reference solution and the test solution were precisely aspirated by 10 µ l respectively, injected into the liquid chromatograph, and measured.

## Chromatogram of test sample

The chromatogram of the test sample showed chromatographic peaks with the same retention time as those of the reference substance, and 8 characteristic peaks corresponding to the reference fingerprint. The similarity was calculated by the characteristic peak. Except peak 7 salvianolic acid B, the similarity between the test sample fingerprint and the reference fingerprint was not less than 0.90 (Supplementary Figure 3)


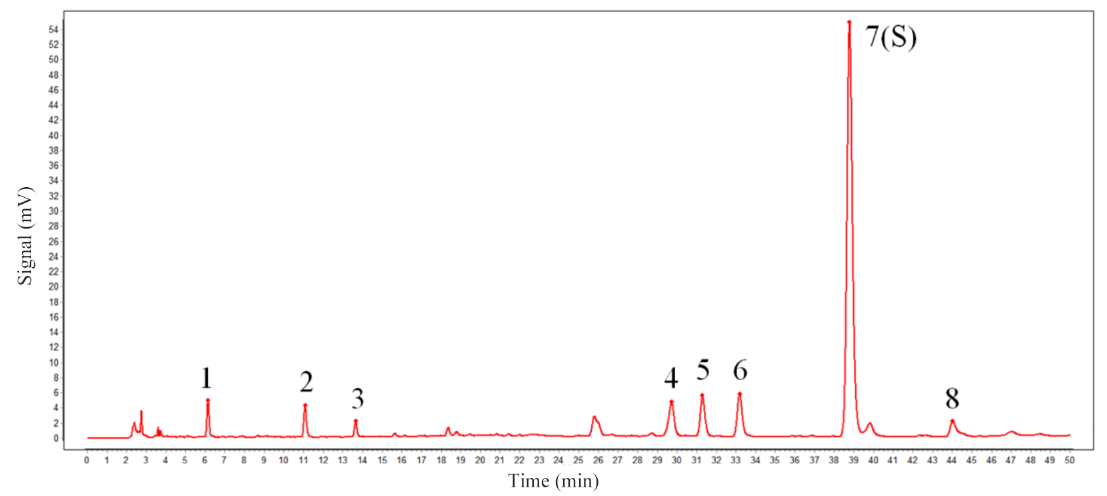
**Supplementary Figure 3.** Contrast characteristics. Peak 1: Danshensu. Peak 2: protocatechuic aldehyde. Peak 3: caffeic acid. Peak 4: salvianolic acid E. Peak 5: rosmarinic acid. Peak 6: Violet oxalic acid. Peak 7 (S): salvianolic acid B. Peak 8: Salvianolic acid L.
